# Stratification of knee osteoarthritis: two major patient subgroups identified by genome-wide expression analysis of articular cartilage

**DOI:** 10.1136/annrheumdis-2017-212603

**Published:** 2017-12-22

**Authors:** Jamie Soul, Sara L Dunn, Sanjay Anand, Ferdinand Serracino-Inglott, Jean-Marc Schwartz, Ray P Boot-Handford, Tim E Hardingham

**Affiliations:** 1 Wellcome Trust Centre for Cell-Matrix Research, Division of Cell-Matrix Biology and Regenerative Medicine, School of Biological Sciences, Faculty of Biology, Medicine and Health, Manchester Academic Health Science Centre, University of Manchester, Manchester, UK; 2 Department of Orthopaedic Surgery, Stockport NHS Foundation Trust, Stockport, UK; 3 Department of Vascular Surgery, Central Manchester NHS Foundation Trust, Manchester, UK; 4 Division of Evolution and Genomic Sciences, School of Biological Sciences, Faculty of Biology, Medicine and Health, Manchester Academic Health Science Centre, University of Manchester, Manchester, UK

**Keywords:** knee osteoarthritis, osteoarthritis, inflammation, disease activity, chondrocytes

## Abstract

**Introduction:**

Osteoarthritis (OA) is a heterogeneous and complex disease. We have used a network biology approach based on genome-wide analysis of gene expression in OA knee cartilage to seek evidence for pathogenic mechanisms that may distinguish different patient subgroups.

**Methods:**

Results from RNA-Sequencing (RNA-Seq) were collected from intact knee cartilage at total knee replacement from 44 patients with OA, from 16 additional patients with OA and 10 control patients with non-OA. Results were analysed to identify patient subsets and compare major active pathways.

**Results:**

The RNA-Seq results showed 2692 differentially expressed genes between OA and non-OA. Analysis by unsupervised clustering identified two distinct OA groups: Group A with 24 patients (55%) and Group B with 18 patients (41%). A 10 gene subgroup classifier was validated by RT-qPCR in 16 further patients with OA. Pathway analysis showed increased protein expression in both groups. PhenomeExpress analysis revealed group differences in complement activation, innate immune responses and altered Wnt and TGFβ signalling, but no activation of inflammatory cytokine expression. Both groups showed suppressed circadian regulators and whereas matrix changes in Group A were chondrogenic, in Group B they were non-chondrogenic with changes in mechanoreceptors, calcium signalling, ion channels and in cytoskeletal organisers. The gene expression changes predicted 478 potential biomarkers for detection in synovial fluid to distinguish patients from the two groups.

**Conclusions:**

Two subgroups of knee OA were identified by network analysis of RNA-Seq data with evidence for the presence of two major pathogenic pathways. This has potential importance as a new basis for the stratification of patients with OA for drug trials and for the development of new targeted treatments.

## Introduction

Osteoarthritis (OA) is a complex joint disease with variable aetiology, symptoms and outcome. It is highly prevalent with major risk factors of ageing, joint trauma and obesity. Although showing no simple pattern of Mendelian inheritance,^1^ OA is estimated to have ~50%genetic contribution and GWAS studies have revealed multiple low risk genes.^2^ OA involves pathological changes in most joint tissues including cartilage, bone, synovium, ligaments and adjunct joint tissues and includes variable development of osteophytes, subchondral bone cysts and sclerosis.^3–5^ The degenerative changes lead to the failure of the joint, which through pain and incapacity causes major morbidity. Progress in the development of disease modifying drugs has been slow and there remains a large unmet clinical need.^6^ Stratification on histopathological features has revealed subgroups of OA, but these have not readily given new insights into the molecular processes of the disease or allowed the development of new drugs.^7^

To develop new treatments, it would help to gain more understanding of OA heterogeneity. New omics technologies offer the possibility to search for different pathogenic mechanisms and thereby identify OA subtypes in an unbiased way. We have used genome-wide sequencing to investigate articular cartilage gene expression. Cartilage was selected as it forms the major load-bearing surface in the joint and it is exposed via synovial fluid (SF) to systemic and local factors within the joint. The pattern of gene expression in OA cartilage therefore reflects intrinsic chondrocyte responses and the influence of extrinsic systemic, synovial, biochemical and biomechanical factors. The analysis reported here is on intact OA cartilage and thus avoids the gene expression changes that occur in damaged cartilage, on which we have previously reported.^8^

This is the largest scale gene expression analysis of cartilage of patients with OA to date and provides new insight into the heterogeneity of OA.

## Methods

### Study design

Intact cartilage from the posterior lateral condyle (PLC) was obtained under Ethics Committee approval with prior informed consent at total knee replacement (TKR) from patients with predominant medial compartmental OA and from non-OA age-matched control samples (online supplementary methods). Histology grading (modified Mankin score), DNA and GAG analysis, extraction of RNA and submission for RNA-Sequencing (RNA-Seq) was as previously described (online supplementary methods).^8–10^

10.1136/annrheumdis-2017-212603.supp1 Supplementary file 1

### RNA sequencing and analysis

RNA sequencing, quality control, read mapping and quantification were performed by standard bioinformatic methods.^11 12^ Fold changes were calculated with DESeq2 with P values adjusted for multiple testing with Benjamini-Hochberg correction. Differentially expressed genes (DEGs) are reported with an absolute fold change of ≥1.5 and a false discovery rate of ≤10%. Unsupervised clustering was carried out (online supplementary methods) with cluster stability and patient fit to each subgroup assessed by silhouette score. DEGs were analysed using Reactome pathway and Cytoscape PhenomeScape app (online supplementary methods).

### Creation of RT-qPCR classifier panel

Shrunken centroid clustering was used (online supplementary methods) to identify the optimum gene set to create a classifier able to distinguish the groups by RT-qPCR.

### Data availability

Fastq files are available to download from ArrayExpress E-MTAB-6266. Code to reproduce the analysis at https://github.com/soulj/OAStratification.

## Results

### Genome-wide expression analysis of intact OA cartilage

Genome-wide expression by RNA-Seq analysis was determined on intact cartilage tissue sampled at TKR for each of 44 patients with OA. All samples were visually intact, with a mean histological score of less than 10 (modified Mankin) as previously reported.^8^ Control age-matched, non-OA cartilage from the same PLC site showed similar modified-Mankin scores, but had slightly higher mean proteoglycan and slightly lower mean DNA content (online supplementary figure 1). Although the non-OA donors were 9 male/1 female, the female was not an outlier in any of the parameters measured. The gene expression determined (RNA-Seq) from patients with OA and non-OA was thus from age-matched full depth cartilage.

**Figure 1:**
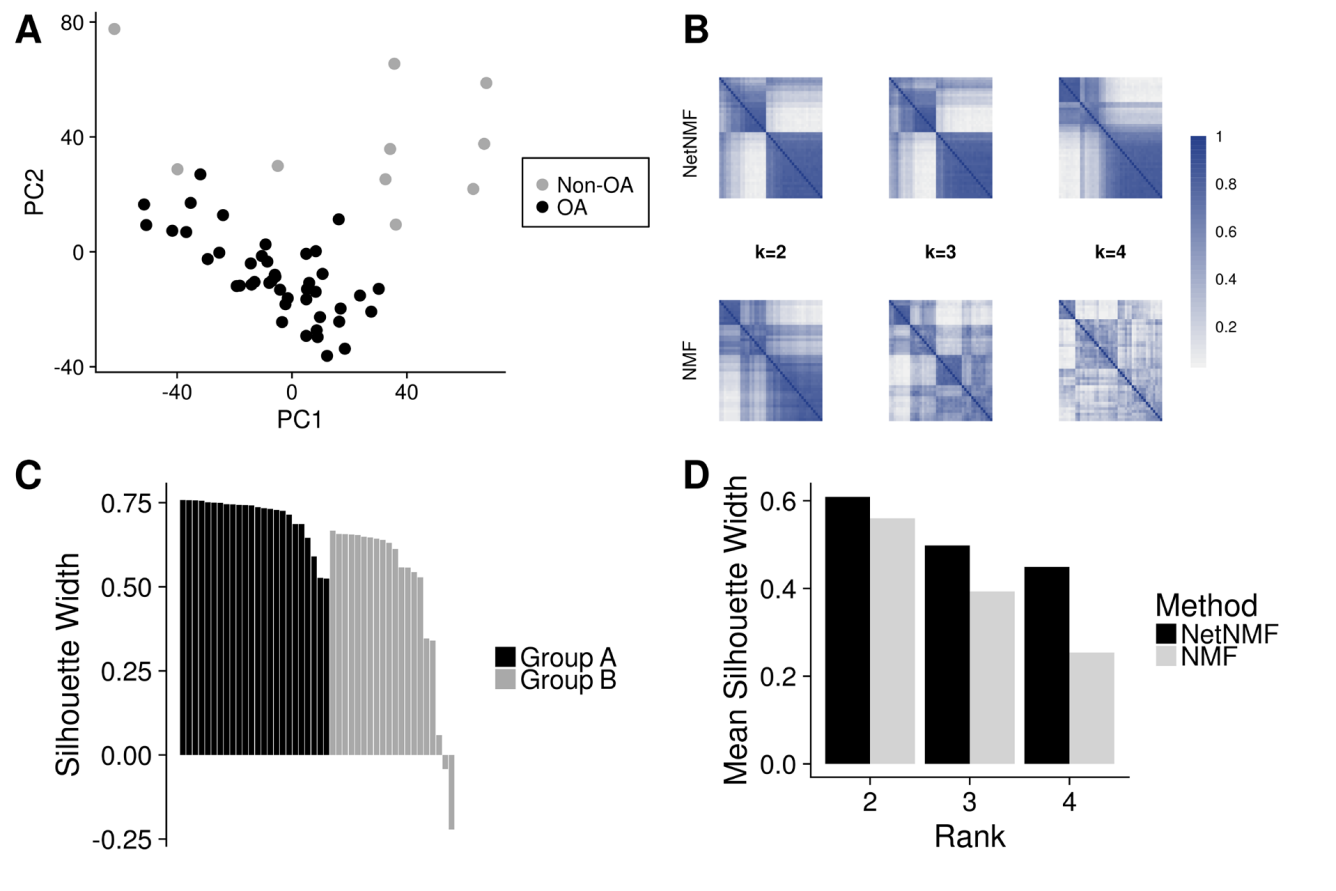
Unsupervised clustering of gene expression from patients with OA. RNA-Seq data from 44 PLC cartilage samples taken at total knee replacement and 10 non-OA PLC samples were normalised and batch effect corrected. Principal component analysis of the data was performed and the first and second (PC1 and PC2) principal components are shown (A). Network-based NMF or standard NMF was used to cluster the patients with OA into subgroups with variable numbers of predefined clusters (k). Patient co-clustering matrices are coloured to show patient pairwise co-occurrence in the same cluster across the 500 clustering runs (B). Silhouette scores were used to assess the optimal number of clusters (C) and the patient fit to a subgroup (D). NMF, negative matrix factorisation; OA, osteoarthritis; PLC, posterior lateral condyle.

The RNA-Seq data were normalised and batch corrected using standard bioinformatic tools (see ‘Methods’ section). After normalisation, principal component analysis of the samples showed some heterogeneity (figure 1A), but the non-OA samples were distinct from the patients with OA and comparison of the results from the 44 patients with OA with the 10 non-OA controls identified 2692 DEGs (online supplementary table 1). The intact OA cartilage from the 44 patients thus showed a broad range of changes in gene expression compared with control non-OA cartilage. These changes also showed major overlap with previous microarray comparisons of expression in OA and non-OA cartilage.^13–15^

10.1136/annrheumdis-2017-212603.supp2 Supplementary file 2

### Stratification of gene expression

The gene expression results from the 44 patients with OA were analysed to search for possible subsets that shared selective changes in expression. Using an unbiased network-assisted, non-negative matrix factorisation (Network NMF) algorithm, which has been demonstrated to yield biologically informative disease clusters, the most stable clustering identified two subgroups (figure 1B and D).^16 17^ Measure of the degree of fit (Silhouette score, see online supplementary methods) revealed strong identity for most patients within each cluster (figure 1C). Only two patients showed no concordance with either group and these were not included in further analysis. The RNA-Seq results thus identified that patients were in two main groups: Group A 55% (n=24) and Group B 41% (n=18) and these showed no association with histological grade, sex, age, body mass index or pain scores (VAS, KOOS) (online supplementary figure 2).

### Classification of new patient samples

We next sought to establish if we could identify each group by RT-qPCR analysis of the expression of a small panel of genes. Using a shrunken centroid classifier approach, we identified an optimal set of 10 genes, whose expression discriminated between the groups. The gene selection predicted from RNA-Seq was validated by RT-qPCR analysis (online supplementary figure 3A,B) and used to train a SVM classifier with fivefold cross-validation using the known group identities. The expression pattern of these 10 genes was conserved in a validation cohort of 16 patients (online supplementary figure 3C) and the RT-qPCR classifier showed an area under the curve of 0.92 in predicting the patient groups compared with the full RNA-Seq analysis.

### Pathways activated in Group A and Group B relative to non-OA

**Figure 2:**
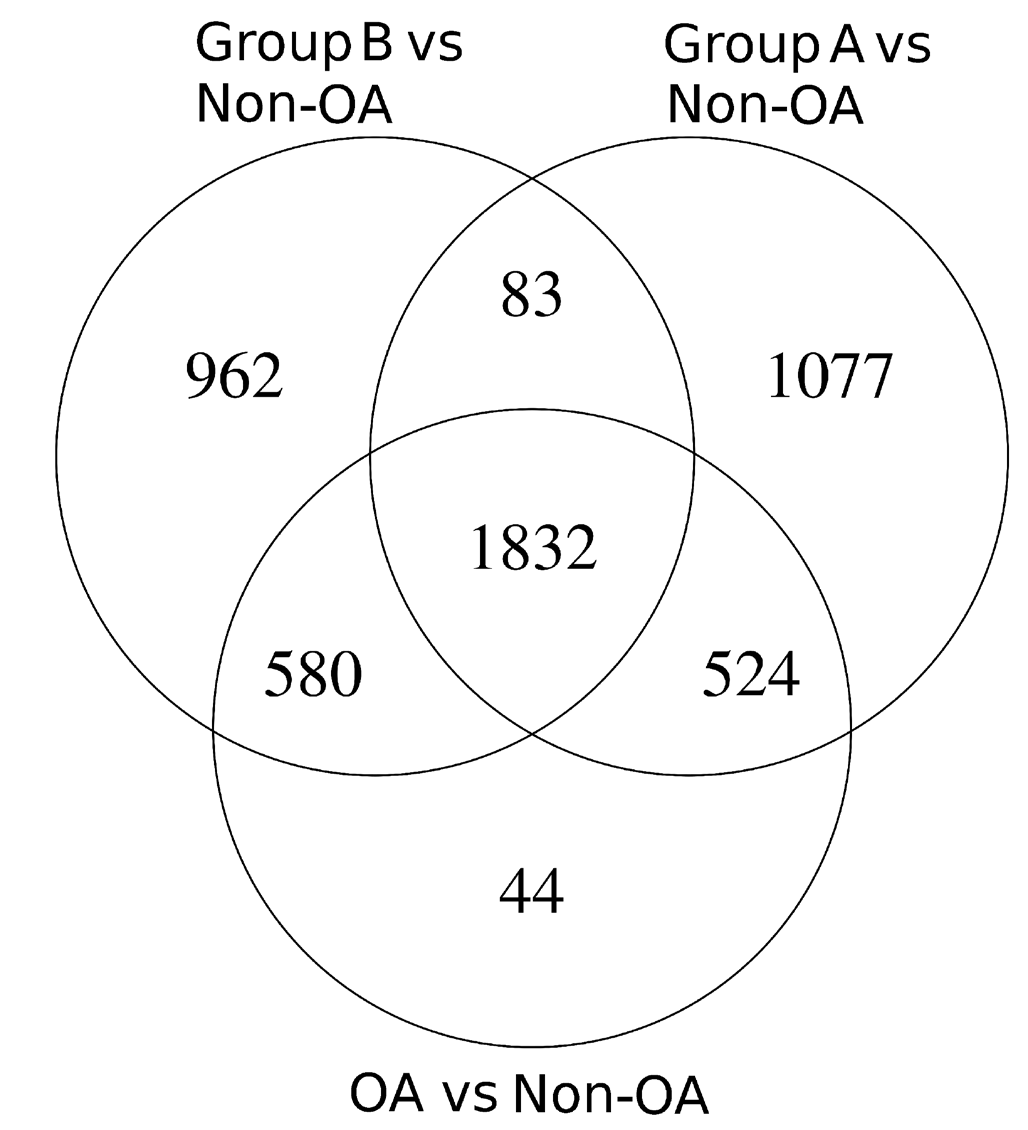
Differentially expressed genes in OA Group A and Group B. Differentially expressed genes (**≥**1.5 FC and ≤0.1 adjusted P values) were determined for each OA Group A (n=32) and Group B (n=26) compared with patients with non-OA (n=10). OA, osteoarthritis.

In the full gene expression analysis from 60 patients, there were 2980 genes that were differentially expressed (DEGs) compared with non-OA (online supplementary table 2). However, when the results were divided into Group A and Group B and each compared separately to non-OA, there were an additional 2122 DEGs detected, with 1077 of those distinctive to Group A and 962 distinctive to Group B (figure 2 and online supplementary table 3). Splitting the patients into two groups thus identified many more gene changes specific to OA.

10.1136/annrheumdis-2017-212603.supp3 Supplementary file 3

10.1136/annrheumdis-2017-212603.supp4 Supplementary file 4

For the initial analysis, we used an unbiased Reactome pathway approach to compare DEGs in each group with non-OA cartilage (online supplementary table 4). This identified in Group A changes in chemokine signalling, inflammasome activation, changes in glycosaminoglycan synthesis, toll receptor activation and innate immune responses, whereas Group B included complement regulation, Wnt signalling, eicosanoid receptor signalling and syndecan interactions (table 1).

10.1136/annrheumdis-2017-212603.supp5 Supplementary file 5

**Table 1:** Reactome pathway analysis

Pathway	Adj. P value
Group A vs non-OA	
EukAryotic translation elongation	6.2e–63
Extracellular matrix organisation	7.3e–13
Chemokine receptors bind chemokines	0.00615
MyD88 deficiency (TLR2/4)	0.04693
Glycosaminoglycan metabolism	0.00584
Inflammasomes	0.01242
Group B vs non-OA	
Eukaryotic translation elongation	1.6e–69
Extracellular matrix organisation	4.3e–12
Negative regulation of TCF-dependent signalling by WNT ligand antagonists	0.00707
Regulation of complement cascade	0.00758
Eicosanoid ligand-binding receptors	0.00758
Syndecan interactions	0.01795
Group B vs Group A	
Extracellular matrix organisation	9.2e–11
Chemokine receptors bind chemokines	8.9e–08
Complement cascade	0.00012
G alpha (i) signalling events	0.00036
Adaptive immune system	0.00068

A selection of highly significant differentially regulated Reactome pathways between OA Group A (n=32) and Group B (n=26) and non-OA (n=10).

OA, osteoarthritis.

Reactome pathways also showed that a major feature common to both groups was multiple changes in ribosomal protein expression (table 1 and online supplementary table 4). This may have two core origins: first, reflecting a large increase in protein expression in OA chondrocytes as part of a disease response^16^ and second, a change in chondrocyte differentiation that results in quite distinct patterns of altered gene expression.^18^

The second major change was in matrix protein genes that were highly expressed. In the 100 most expressed genes in non-OA cartilage, there were 27 matrix genes, but this increased to 37 matrix genes in Group A and 39 in Group B. The results showed a large increase in matrix protein gene expression in both patient groups, but as detailed below each group showed a different pattern of changes. Several growth factors with anabolic effects (IGF2, FGF1, 2 and 18, TGFβ1, 3 and GDF5, 10 and BMP6) were increased in both groups, potentially supporting the increased and altered pattern of protein expression (online supplementary table 3). A list of the major pathways common to both groups compared with non-OA is shown in online supplementary table 4.

### Group A and Group B show altered patterns of matrix protein gene expression

Group B showed changes reflecting a switch to less chondrogenic genes with reduced expression of the major chondrocyte transcription factor, SOX9 and SIRT1, which is a supporter of chondrogenesis and reduced expression of the matrix genes, aggrecan (ACAN) and type IX collagen (COL9A2/3)^19 20^ (online supplementary table 3 and 5). In contrast the non-chondrogenic genes versican (VCAN) and Type I collagen (COL1A1/2) were increased together with the pericellular matrix collagens, (COL6A1, 2, 3), laminins (LAMA2, A4, B1, C1) and perlecan (HSPG2) (supplementary table 3 and 5).^21^ An altered phenotype in Group B is also suggested by increased expression of transcription factors associated with an osteogenic phenotype, including osterix and RUNX2 (online supplementary table 5). Together these results suggest the chondrocytes in Group B patients are active in a less chondrogenic pathway of remodelling. In contrast, Group A showed increased expression of major cartilage collagens Type II, Type V and Type IX and Type XI and less expression of Type I (online supplementary table 3).

10.1136/annrheumdis-2017-212603.supp6 Supplementary file 6

### Pathway analysis comparing Group A with Group B

**Figure 3:**
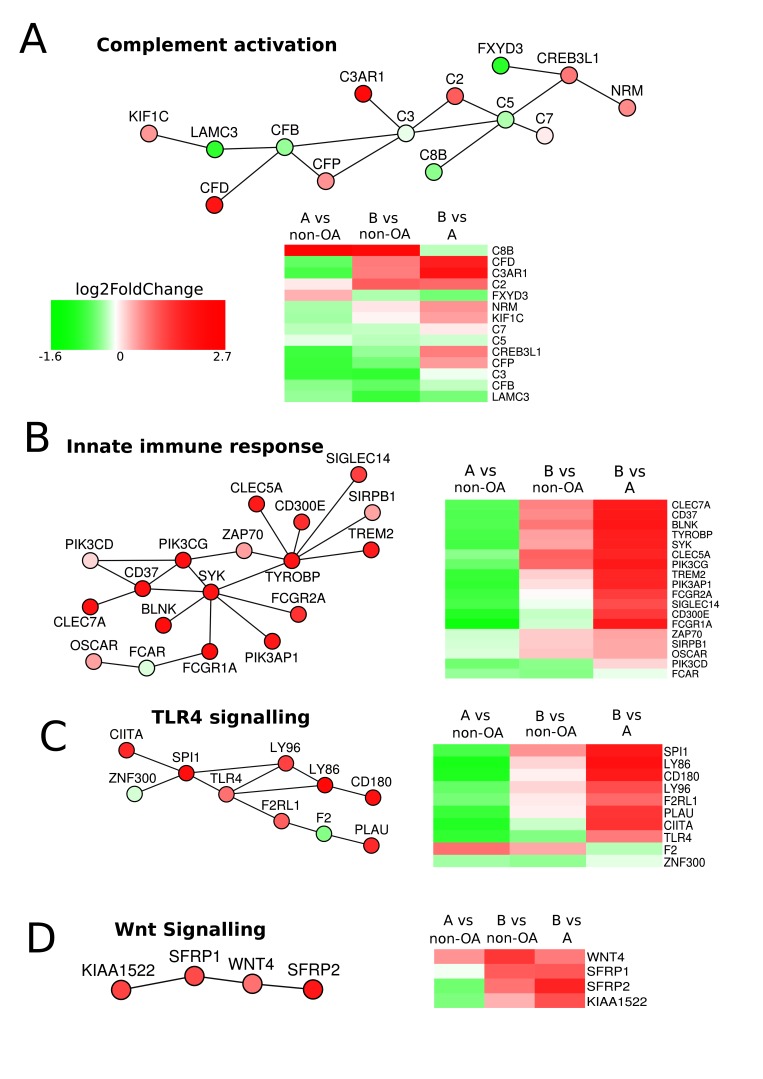
PhenomeExpress analysis of OA Group A and Group B. Differential gene expression data between Group A (n=32) and Group B (n=26) were analysed with the PhenomeExpress algorithm to identify dysregulated subnetworks using known disease gene associations. Nodes are coloured by fold change. Heatmaps show the gene expression between Group A, Group B and non-OA for each subnetwork.

To investigate further the differences between the two groups, we applied a network-based PhenomeExpress analysis incorporating data from published disease-related studies.^22 23^ This technique identifies gene-sets that have known direct molecular interactions and scores them based on the total differential expression of the gene-set. Activities distinguishing the two groups included oxidative stress, innate-immune responses, Wnt signalling, chemokine signalling, apoptotic threshold and calcium regulation (figure 3; online supplementary figure 3 and supplementary table 6). The heatmaps of this analysis shows downregulation of genes in many pathways in Group A compared with non-OA. Where such pathways are protective to cartilage homeostasis, Group A could be more susceptible to other adverse changes that may be metabolic and/or mechanical and caused by extrinsic/environmental or intrinsic/genetic factors.

10.1136/annrheumdis-2017-212603.supp7 Supplementary file 7

### Changes in circadian rhythm and TGFβ signalling

Circadian clock mechanisms have recently been identified as an important pathway in regulating cartilage matrix homeostasis. As the circadian regulation of genes controls a 24 hours cycle of coordinated matrix protein synthesis and degradation,^24^ any change in amplitude or phase may contribute to a loss of matrix integrity. Circadian disruption by genetic deletion of the regulator BMAL1 in mouse cartilage results in an early onset joint degeneration.^20 24^ In our expression results, BMAL1 (ARNTL) and its binding partner NPAS2 were reduced in both Groups compared with non-OA, whereas in contrast, CRY1/2 (members of the circadian oscillator complex) were increased (table 2A). This change in ratio suggests an imbalance in clock control and although the timing of cartilage sampling was not strictly controlled, it was generally early in the same quarter (09:00–15:00 hours) of the 24 hours cycle and the ratio is less variable than the absolute levels of expression (24). There was also reduced expression of other genes involved in circadian regulation (table 2A) including the transcriptional repressor Rev-ErbA (NR1D1).^25^ Consequences of the loss of circadian rhythm involve the downregulation of transcriptional regulators such as NFATC1/2.^26 27^ In our data, the downstream targets NFATC1/2 were increased in expression in both groups, which suggests their expression is maintained independent of the circadian changes. Clock disruption may act through other major pathways and TGFβ signalling was shown to be affected by BMAL or NR1D1 knockdown in cultured human articular chondrocytes, which resulted in altered SMAD signalling and increased TGFB3 and the receptor TGFBR1 expression.^26 27^ As these were increased in expression in both patient groups, upregulated activity in TGFβ signalling pathways may account for the increased expression of target genes, including cartilaginous and non-cartilaginous matrix proteins (table 2).^28^

**Table 2:** Expression changes in circadian clock related genes in  patient Groups A and B; 2B—expression changes in mechanosensitive ion channels and cytoskeletal proteins in patient Groups A and B

	Name	Group A fold change	Group B fold change
(A) Gene			
ARNTL	Bmal1 primary clock oscillator	−1.67	−1.88
NPAS2	Transcription factor activator interacts with Bmal1	−1.64	−1.85
CRY1	Inhibitor of Bmal1/NPAS2 as part of clock oscillator	+1.33	+1.37
PER1	Inhibitor of the core clock oscillator	−1.50	−1.87
NR1D1	Rev-Erbα nuclear hormone receptor, negative regulator of Bmal1 transcription	−1.72	−1.90
BHLHE40	DEC1, Basic helix-loop-helix Target of clock	NS	−1.35
RORC	Nuclear hormone receptor transcr. factor, positive regulator of Bmal1 transcription	−1.40	−2.15
DBP	Transcription factor activating clock target genes via D-box cis elements	+2.39	+1.95
NFIL3	Transcription factor suppressing clock target genes via D-box cis elements	−2.29	−2.67
NFATC1	Component of DNA binding transcription complex	+1.49	+1.30
NFATC2	Component of DNA binding transcription complex	+3.02	+3.33
(B) Ion channels			
TRPV4	Mechanosensitive Ca++channel	+2.28	+1.59
AQP1	Aquaporin, water channel	NS	+3.28
ANO1	Voltage gated Ca++activated Cl- channel	+2.27	+3.15
KCNA1	K+voltage gated channel	+1.61	+1.63
KCNQ5	K+voltage gated channel	+1.53	+1.57
KCNS3	K+voltage gated channel modifier	NS	+2.53
KCNK6	K+channel	NS	+1.31
KCNN3	Ca++activated K+channel	+1.71	+2.64
KCNN4	Ca++activated K+channel	NS	+2.86
KCNMB4	Ca++activated K+channel subunit 4	NS	+1.58
KCTD12	K+channel tetramerisation domain containing 12	NS	+1.96
SCNNIA	Non-gated Na+channel	−3.46	−4.94
Cytoskeleton			
ACTA2	Alpha smooth muscle actin	−2.86	+1.81
VIN	Vinculin cytoskeletal protein	NS	+1.37
TMSB4X	Thymosin B4 cytoskeletal protein	NS	+1.87
EZR	Ezrin-Plasma mem-cytoskeletal link	NS	+2.08
TPM4	Tropomyosin 4 actin binding	NS	+1.36
MYH9/11	Smooth muscle myosin heavy chain, contractile protein	9—NS 11—NS	+1.32 +2.30

GWAS, genome-wide association study.

### Mechanical load-related changes

Joint cartilage has a prime load bearing role. Repetitive loading is necessary for maintaining cartilage function and whereas excess overload can cause damage, joint disuse also leads to atrophic changes. Chondrocytes therefore have mechanosensitive homeostatic mechanisms that regulate the assembly and maintenance of cartilage matrix.^29^

The increases in less chondrogenic genes in Group B may reflect a response to a changed pattern of mechanical stress across the joint causing altered loading on chondrocytes.^30 31^ Mechanosensors, ion channels, calcium control, water balance and cytoskeletal tensing are all implicit in chondrocyte load responses,^29 31 32^ and these show a spectrum of changes, which are more pronounced in Group B. The water channel AQP1 and the main calcium regulated ion channel in chondrocytes TRPV4 are strongly increased in Group B.^32 33^ Also increased in Group B are other calcium-activated potassium channels including KCNN3, 4,^34^ the voltage sensitive calcium activated chloride channel ANO1 and potassium channels KCNQ5, KCNS3 and KCTD12, KCNMB4 (table 2B). Cytoskeletal proteins in Group B also showed increased alpha smooth muscle actin (ACTA2) and increases in contractile proteins, scaffold and membrane proteins, which together suggest chondrocytes are exposed to greater shear stress and deformation (table 2B).^35^ Group A also showed increased expression of TRPV4, but low or no increase in these other ion channels and cytoskeletal proteins.

### Inflammation and proteases

Inflammation has been proposed as a contributor to OA progression.^36–38^ Although the gene expression reported here is from cartilage chondrocytes, the pattern of expression would include responses to inflammatory mediators from surrounding tissues and SF that penetrate cartilage. However, in the intact OA cartilage sampled in both groups exhibited very low expression and no differential expression of IL1α, β, TNF, IL6 and OSM and also of CCL2 (MCP-1), CXCL9 (MIG) and CXCL10 (IP10) (online supplementary table 3). Furthermore, the alarmins S100A8/9 (MRP8/14), which are linked to activation of the inflammatory cascade,^39^ were also less expressed in both OA groups. The expression of NFκΒ complex proteins, such as the kinase activator (IKK- β) and the inhibitory binding partner IKB-β were less expressed in both groups and IKK-γ (NFκB essential modulator) was little changed compared with non-OA. There was thus little evidence of classic inflammatory pathway activation in the intact OA cartilage sampled or of activation by factors from SF.

A range of inflammatory cytokines have been detected in OA SF, although only at low concentration (0–20 pg/mL).^40 41^ As several growth factors are increased in expression in both OA groups (see above) with the potential to reverse the response of chondrocytes to inflammatory cytokines,^42 43^ it appears likely that any potential effect of cytokines at the concentrations present in SF on chondrocyte gene expression may be overcome by endogenous growth factors. As knee OA is a slowly progressive condition, more evidence is needed from earlier disease stages to show when inflammatory mediators are expressed in chondrocytes and/or are present in SF at high enough concentrations to contribute to cartilage pathology.

The expression of matrix proteinases showed that a mixed pattern of responses with gelatinase (MMP9) and collagenase (MMP13) both increased and more strongly so in Group B, whereas stromelysin (MMP3) and the aggrecanases (ADAMTS4, 5) were downregulated in both groups. The only matrix proteinase highly expressed by chondrocytes was the serine proteinase HTRA1 which was increased in both groups (online supplementary table 3).

### Integration with other studies

In a previous knee OA study, Fernández-Tajes *et al* reported DNA methylation analysis on cartilage from 25 patients and identified a small subgroup that differed from the rest. Microarray analysis on a separate group of patients identified 47 significantly altered genes that distinguished the small group and were directly correlated with the methylation analysis.^44^ Selecting 9 of the 47 genes, they showed by RT-qPCR these could identify the small subgroup. Comparison of the expression results of these 47 genes with our RNA-Seq analysis showed major overlap (online supplementary table 7) and showed that their panel of nine genes were also all differentially expressed between our Group A and Group B (see supplementary figure 4). These results suggest that the Group B identified in our study corresponds to the small group identified using an entirely different approach by Fernández-Tajes *et al.* 44

10.1136/annrheumdis-2017-212603.supp8 Supplementary file 8

From their results, Fernández-Tajes *et al* concluded that this small subset showed evidence of inflammation and reduced matrix protein synthesis. A further analysis based on GO annotations of gene expression identified inflammatory response, leucocyte activation, cytokine production and chemokine activity, as factors more enhanced in the subset. Our conclusions from Reactome pathway analysis detected some similar changes comparing Group B with Group A (table 1); however, from the more detailed analysis possible with genome-wide expression data across 60 patients, we find strong evidence of increased matrix protein expression in both our patient groups and little evidence of classical inflammatory activation. Our analysis thus agrees with the group identification of Fernández-Tajes *et al* 44, but differs in the conclusions from the pathway analysis.

### Potential biomarkers in synovial fluid

From our gene expression analysis, it was possible to predict 478 secreted proteins (online supplementary table 8) differentially expressed between the two groups that may be released from the articular cartilage and therefore detectable in SF. Many of the 478 secreted proteins are not specific to cartilage and may enter SF from other joint tissues and from the circulation. The differences in their gene expression may also not correlate well with their concentration in SF. However, among these proteins, some may form potential biomarkers that could enable the identity of the two groups to be determined by SF analysis and leading candidates include several proteins already detected in SF and cartilage explant culture media (table 3). Large scale proteomic analysis of OA SF correlated with the differential expression in patients’ cartilage of the classifier set of 10 genes may enable a select group of proteins to be identified whose combination of concentrations in SF distinguishes patients in the two groups. This would make more accessible the use of these results to stratify patients with OA.

10.1136/annrheumdis-2017-212603.supp9 Supplementary file 9

**Table 3:** Chondrocyte secreted proteins differentially expressed in Group A and Group B

Gene	Protein	Group A fold increase	Group B fold increase
WIF1	Wnt inhibitor factor 1	1.82	
S100A1	S100 Ca++binding protein	1.77	
S100B	S100 Ca++binding protein	1.72	
C4BPA	C4 binding protein A	1.73	
GPX3	Glutathione peroxidase 3	1.62	
ADAMTSL2	AdamTS-like 2, microfibril assembly	1.55	
SCRG1	Stimulator of chondrogenesis	1.48	
CHAD	Chondroadherin	1.47	
MGP	Matrix Gla protein	1.47	
SERPINF1	Pigment epithelium derived factor		3.60
RNASE1	Pancreatic style secretory RNAse		3.56
SPARCL1	Sparc-like protein 1 (hevin)		3.11
POSTN	Periostin		2.79
PLTP	Phospholipid transfer protein		2.26
IGFBP4	IGF binding protein 4		2.23
SFRP1	Secreted Frizzled related protein 1		2.19
CLEC3B	Tetranectin		2.07
TGFBI	TGF beta induced protein		2.03
DKK3	Dickkopf 3, Wnt inhibitor		2.00
TNC	Tenascin C		1.80
TIMP3	Tissue inhibitor of metalloproteinases 3		1.67
CRTAC1	Cartilage acidic protein 1		1.55
ASPN	Asporin (serine leucine-rich matrix protein)		1.47

## Discussion

A major finding of this study was that the pattern of gene expression in knee OA cartilage falls into two clear groups. The cartilage focused on is from an undamaged site. The results do not therefore reflect different degrees of tissue damage, but are intrinsic to OA cartilage in the two groups. The design of this study also offers an advance over previous microarray comparisons of OA and non-OA cartilage, by using RNA-seq with more complete genome wide coverage and greater dynamic range and by carrying out the study on a scale large enough to assess heterogeneity among patients. The results showed little evidence of induced inflammatory responses in each of 60 patients, which suggests that at this late stage of disease, the effect of local inflammation is not a common or persistent factor. This is largely in agreement with other studies that have noted little evidence for inflammatory cytokine activity in late OA.^40 45^

The common changes in circadian regulators in both OA groups suggest that a decline in clock regulation may be an important factor that adversely affects the fundamental mechanisms of cartilage homeostasis and, in the long term, this may compromise tissue integrity. More detailed studies are needed to substantiate this and to understand the circadian impact on other major biological and mechanical signalling pathways and how they relate to the major increases and different patterns of matrix protein gene expression. A possible cause for the matrix protein response in Group B being less chondrogenic is suggested by a whole set of gene expression changes linked to altered mechanical demands, such as increased tissue shear. Thus, there was increased expression of key mechanoreceptors, in calcium signalling, in associated ion channels, in cytoskeletal organisers, in cell matrix interface and pericellular proteins, which all have links to mechanotransduction. The altered mechanical environment may result in a switch in chondrocyte phenotype, which results in a less chondrogenic response. The different pattern of matrix expression in the two groups would predict altered cartilage matrix properties, but it will require measurements of tensile and compressive stiffness to determine the different biomechanical changes in the two groups and to show if these changes predict different rates of progression of cartilage damage.^30 46^

Many failed clinical trials in knee OA have possibly benefited some patients, but had poor success overall and have not delivered clear benefit above placebo within the time frame of the trial.^47–49^ The results in this study identify a metabolic basis for stratifying patients into two groups, which are based on major differences in gene expression. While these changes are specific to chondrocytes in cartilage they may also reflect responses to mechanical signals and biological mediators from across the joint. This new route to OA stratification is potentially important as it suggests that the different patterns of gene expression in the two groups reflect different metabolic activities and raises the possibility that the responses of the two groups may be susceptible to different modes of intervention.^47^ To realise any practical benefit from this analysis requires further validation and testing. The identification of the classifier panel of genes and potential biomarkers in SF provide tools to facilitate progress, but new investigations are needed to show any predictive value and establish if stratifying patients on this molecular basis can help distinguish responders and non-responders and enable preselection of patients that respond more consistently to new targeted treatments.
