# Supplementary material for: Stratification of knee osteoarthritis: two major patient subgroups identified by genome-wide expression analysis of articular cartilage
Source: Ann Rheum Dis. 2017 Dec 22;77(3):423. doi: 10.1136/annrheumdis-2017-212603 (PMC5867416; doi:10.1136/annrheumdis-2017-212603)
Supplement: Supplementary file 1 [file annrheumdis-2017-212603supp001.pdf]

Supplementary methods

Study design

Articular cartilage was obtained under Ethics Committee approval with prior written informed consent at total knee replacement (TKR) for OA patients (IRAS 114697). The discovery cohort comprised 44 OA patients (21M and 23F, age range 63-85 years, mean age 72). Cartilage was obtained from a further 16 OA patients that formed the validation cohort (12M and 4F, age range years 63-81, mean age 72). Cartilage was isolated from the posterior lateral condyle (PLC) which matched the criteria for intact cartilage previously published.[1] Comparable PLC samples from 10 non-OA joints (9M and 1F, age range 55-86, mean age 64) were obtained from above knee amputations of patients with peripheral vascular disease and no history or clinical sign of OA, joint disease, or joint trauma. KOOS and VAS pain scores were routinely collected in the clinical setting.[2,3] All OA patient donors analysed had predominant medial compartment OA, which in our clinical setting was >90% of all patients at TKR. Because of possible compromise to the PLC samples, any patient joints with predominant lateral compartment changes were not taken for analysis. Significant differences between patient phenotypes were tested using Mann–Whitney U or Chi-squared tests. Extracted RNA was submitted for RNA-Sequencing (RNA-Seq) as previously described.[1]

Histology and Tissue Analysis

De-calcified osteochondral samples were dehydrated in graded ethanol (Fisher Scientific) and immersed in xylene (Sigma–Aldrich). Samples embedded in paraffin wax were cut into sections (5µm thickness) and stained with 0.1% safranin O-fast green for histological grading using a modified Mankin score, as previously described.[1] The proteoglycan (GAG) content of cartilage tissue from the PLC and DMC was determined after overnight digestion in papain at 60 C using the dimethylmethylene blue (DMMB) assay with absorbance read at 570 nm and the total DNA was determined on the same digest using Hoechst method.[4,5]

RNA extraction

Total RNA was extracted from each patient sample of 200 to 400 mg of cartilage using TRIzol (LifeTechnologies) reagent and homogenisation (Braun Mikrodismembrator) following freezing in liquid nitrogen. The RNA was purified using RNeasy Qiagen clean-up columns (Qiagen) and for sequencing had a RIN score of >6 (2200 TapeStation, Agilent Technologies).

RT-qPCR

cDNA was synthesised from 0.5 to 1µg of total RNA using MLV reverse transcriptase and random hexamers (Life Technologies). For RT-qPCR analysis primer details are listed in Supplementary Table 9. Gene expression was normalised to an average of CANX (calnexin), CSDE1 (cold shock domain containing E1) and EIF4G2 (eukaryotic translation initiation factor 4 gamma 2). Reference genes were chosen by identifying the least variable genes between the OA patients in our data.

Relative gene expression levels were determined using the $2^{-\Delta\Delta Ct}$ analysis method.[6] Differences in expressed genes were identified using the Mann–Whitney U test where P-values ≤0.05 were considered significant.

RNA sequencing

RNA-Seq analysis using Illumina HiSeq 2500 was performed on fresh cartilage from the posterior lateral condyle of OA patients and the 10 non-OA controls. Strand specific RNA-Seq libraries were generated and yielded a mean of 27 million reads per sample. Quality control was performed with FastQC. Read mapping and gene level quantification were performed using kallisto with Ensembl 38, release 39 giving a mean mapping rate of 92%.[7] Tximport was used to summarise the transcript level counts to gene level counts.[8] For unsupervised clustering, the reads were normalised and variance stabilised with DESeq2, then batch effect normalised with pSVA, which is suited for subsequent unsupervised clustering.[9,10] Fold changes and p-values were calculated with DESeq2 taking into account the batch origin of the samples. The resulting p-values were adjusted for multiple testing with Benjamini–Hochberg (BH) correction. Differentially expressed genes (DEGs) were defined as genes with an absolute fold change of ≥1.5 and adjusted p-values ≤0.1.

Network NMF

The human ConsensusPathDB (v30) network of experimentally derived protein-protein interactions was downloaded and ID conversion performed using BioMart[11,12] An adjacency matrix of a nearest neighbours network derived from the graph Laplacian of an influence distance matrix was created.[13] Network NMF was implemented as a model in the NMF package framework within R.[14] The parameter lambda which regulates the relative influence of the network on the clustering solution was found to produce stable results within a reasonable range as previously reported. A final lambda value of 25 was used. For comparison, standard NMF with the Lee algorithm was performed using the same expression data.[15]

Consensus clustering with random sampling of 90% of the patients and 90% of the genes each run was performed.[16] This procedure was repeated 500 times and clustering outcomes were kept as a co-clustering matrix which records the frequency of each patient pair observed with membership in the same subtype, over all clustering runs in which both patients of the pair were sampled. The final clustering solutions were produced by hierarchical clustering of these co-clustering matrices. The number of subgroups (k) was trialled from k=2-4. The mean silhouette score were used to assess cluster stability and the patient fit to a subgroup.[17] The silhouette score was calculated using the euclidean distance. For patient *i* assigned to subgroup *j* the silhouette score of that patient *S(i)* was calculated as follows:

For a patient sample *i*

Let *a(i)* be the average dissimilarity to all other patients in subgroup *j*

Let *b(i)* be the minimum, average dissimilarity of i to the patients of a subgroup of which i is not a member.

Then:

$S(i) = b(i)-a(i) / max\{a(i),b(i)\}$

S(i) therefore lies between 1 and -1 which indicate strong cluster similarity and dissimilarity respectively. The mean silhouette score for each subgroup was calculated as a measure of subgroup stability. Those patients showing poor association to their assigned cluster (negative silhouette score) were removed from subsequent analysis.

Reactome pathway analysis

Differentially expressed genes were used with the R package GOSeq to identify dysregulated Reactome pathways.[18,19] Pathways with Benjamini–Hochberg (BH) corrected p-values of ≤0.05 were regarded as significant.

PhenomeExpress sub-network identification

The Cytoscape PhenomeScape app was used to identify groups of interacting differentially expressed genes related to OA phenotypes.[20] The default human network and PhenomeExpress settings were used with a size parameter of 7. Phenotypes relevant to OA were chosen from the UberPheno ontology (MP:0003436, HP:0001387, MP:0003724, HP:0002758).

Creation of RT-qPCR panel

The Bioconductor package pamr was used with default settings to perform shrunken centroid clustering with the normalised RNA-Seq data and the identified patient subgroup labels to find the minimal gene set required to accurately classify the samples.[21] The RNA-Seq data was further filtered to keep only strongly expressed genes (median count 5000 reads across all the samples) so to allow robust RT-qPCR quantification. 5-fold cross-validation was performed using a nearest centroid classifier and the smallest gene set that provided the highest class prediction accuracy compared with the classifications made by the complete RNA-Seq dataset was used to inform the gene panel selection.

Classifiers for RNA-Seq and RT-qPCR

The R package caret was used to train support vector machine (SVM) classifiers.[22] The parameter c was tuned by 100 repeats of 5-fold cross-validation. The normalised, batch effect corrected RNA-Seq data and the RT-qPCR $2^{-\Delta\Delta Ct}$ values were used to train the classifiers and subsequently used to predict the unknown samples using each classifier. The R pROC package was used to calculate the AUC for the ROC of the RT-qPCR predictions to the RNA-Seq classifier predictions.[23]

Identification of secreted proteins

UniProt annotations for secreted proteins and proteins identified through synovial fluid/cartilage explant media proteomics studies were cross-referenced with the differentially expressed genes (absolute fold change of ≥1.3 and adjusted p-values ≤0.1) detected between the OA Groups.[24]

1 Dunn SL, Soul J, Anand S, *et al.* Gene expression changes in damaged osteoarthritic cartilage identify a signature of non-chondrogenic and mechanical responses. *Osteoarthr Cartil* 2016;:1–10. doi:10.1016/j.joca.2016.03.007

2 Roos EM, Lohmander LS. The Knee injury and Osteoarthritis Outcome Score (KOOS): from joint injury to osteoarthritis. *Health Qual Life Outcomes* 2003;**1**:64. doi:10.1186/1477-7525-1-64

3 Hawker GA, Mian S, Kendzerska T, *et al.* Measures of adult pain: Visual Analog Scale for Pain (VAS Pain), Numeric Rating Scale for Pain (NRS Pain), McGill Pain Questionnaire (MPQ), Short-Form McGill Pain Questionnaire (SF-MPQ), Chronic Pain Grade Scale (CPGS), Short Form-36 Bodily Pain Scale (SF. *Arthritis Care Res (Hoboken)* 2011;**63**:S240–52. doi:10.1002/acr.20543

4 Hoemann CD. Molecular and biochemical assays of cartilage components. *Methods Mol Med* 2004;**101**:127–56. doi:10.1385/1-59259-821-8:127

5 Kim YJ, Sah RLY, Doong JYH, *et al.* Fluorometric assay of DNA in cartilage explants using Hoechst 33258. *Anal Biochem* 1988;**174**:168–76. doi:10.1016/0003-2697(88)90532-5

6 Livak KJ, Schmittgen TD. Analysis of relative gene expression data using real-time quantitative PCR and. *Methods* 2001;**25**:402–8. doi:10.1006/meth.2001.1262

7 Bray NL, Pimentel H, Melsted P, *et al.* Near-optimal probabilistic RNA-seq quantification. *Nat Biotechnol* 2016;**34**:525–7. doi:10.1038/nbt.3519

8 Soneson C, Love MI, Robinson MD. Differential analyses for RNA-seq: transcript-level estimates improve gene-level inferences. *F1000Research* 2016;**4**:1521. doi:10.12688/f1000research.7563.2

9 Love MI, Huber W, Anders S, *et al.* Moderated estimation of fold change and dispersion for RNA-seq data with DESeq2. *Genome Biol* 2014;**15**:550. doi:10.1186/s13059-014-0550-8

10 Parker HS, Leek JT, Favorov A V, *et al.* Preserving biological heterogeneity with a permuted surrogate variable analysis for genomics batch correction. *Bioinformatics* 2014;**30**:2757–63. doi:10.1093/bioinformatics/btu375

11 Smedley D, Haider S, Durinck S, *et al.* The BioMart community portal: an innovative alternative to large, centralized data repositories. *Nucleic Acids Res* 2015;**43**:W589-98. doi:10.1093/nar/gkv350

12 Kamburov A, Wierling C, Lehrach H, *et al.* ConsensusPathDB--a database for integrating human functional interaction networks. *Nucleic Acids Res* 2009;**37**:D623–8. doi:10.1093/nar/gkn698

13 Hofree M, Shen JP, Carter H, *et al.* Network-based stratification of tumor mutations. *Nat Methods* 2013;**10**:1108–15. doi:10.1038/nmeth.2651

14 Gaujoux R, Seoighe C. A flexible R package for nonnegative matrix factorization. *BMC Bioinformatics* 2010;**11**:367. doi:10.1186/1471-2105-11-367

15 Lee DD, Seung HS. Learning the parts of objects by non-negative matrix factorization. *Nature* 1999;**401**:788–91. doi:10.1038/44565

16 Monti S, Tamayo P, Mesirov J, *et al.* Consensus clustering: A resampling-based method for class discovery and visualization of gene expression microarray data. *Mach Learn* 2003;**52**:91–118. doi:10.1023/A:1023949509487

17 Rousseeuw PJ. Silhouettes: A graphical aid to the interpretation and validation of cluster analysis. *J Comput Appl Math* 1987;**20**:53–65. doi:10.1016/0377-0427(87)90125-7

18 Young MD, Wakefield MJ, Smyth GK, *et al.* Gene ontology analysis for RNA-seq: accounting for selection bias. *Genome Biol* 2010;**11**:R14. doi:10.1186/gb-2010-11-2-r14

19 Croft D. Building models using Reactome pathways as templates. *Methods Mol Biol* 2013;**1021**:273–83. doi:10.1007/978-1-62703-450-0_14

20 Soul J, Dunn S, Hardingham T, *et al.* PhenomeScape: a Cytoscape app to identify differentially regulated sub-networks using known disease associations. *Bioinformatics* 2016;:btw545. doi:10.1093/bioinformatics/btw545

21 Tibshirani R, Hastie T, Narasimhan B, *et al.* Diagnosis of multiple cancer types by shrunken centroids of gene expression. *Proc Natl Acad Sci* 2002;**99**:6567–72. doi:10.1073/pnas.082099299

22 Kuhn M. Building Predictive Models in R Using the caret Package. *J Stat Softw* 2008;**28**:1–26. doi:10.1053/j.sodo.2009.03.002

23 Robin X, Turck N, Hainard A, *et al.* pROC: an open-source package for R and S+ to analyze and compare ROC curves. *BMC Bioinformatics* 2011;**12**:77. doi:10.1186/1471-2105-12-77

24 Dimmer EC, Huntley RP, Alam-Faruque Y, *et al.* The UniProt-GO Annotation database in 2011. *Nucleic Acids Res* 2012;**40**:D565-70. doi:10.1093/nar/gkr1048

Supplementary Figure 1: Patient statistics

Modified Mankin score of the articular cartilage (**A**), the GAG (**B**) and DNA content of the cartilage tissue (**C**) obtained from the PLC from OA (*n*=60) and non-OA (*n*=10) patients used for RNA-Seq analysis.

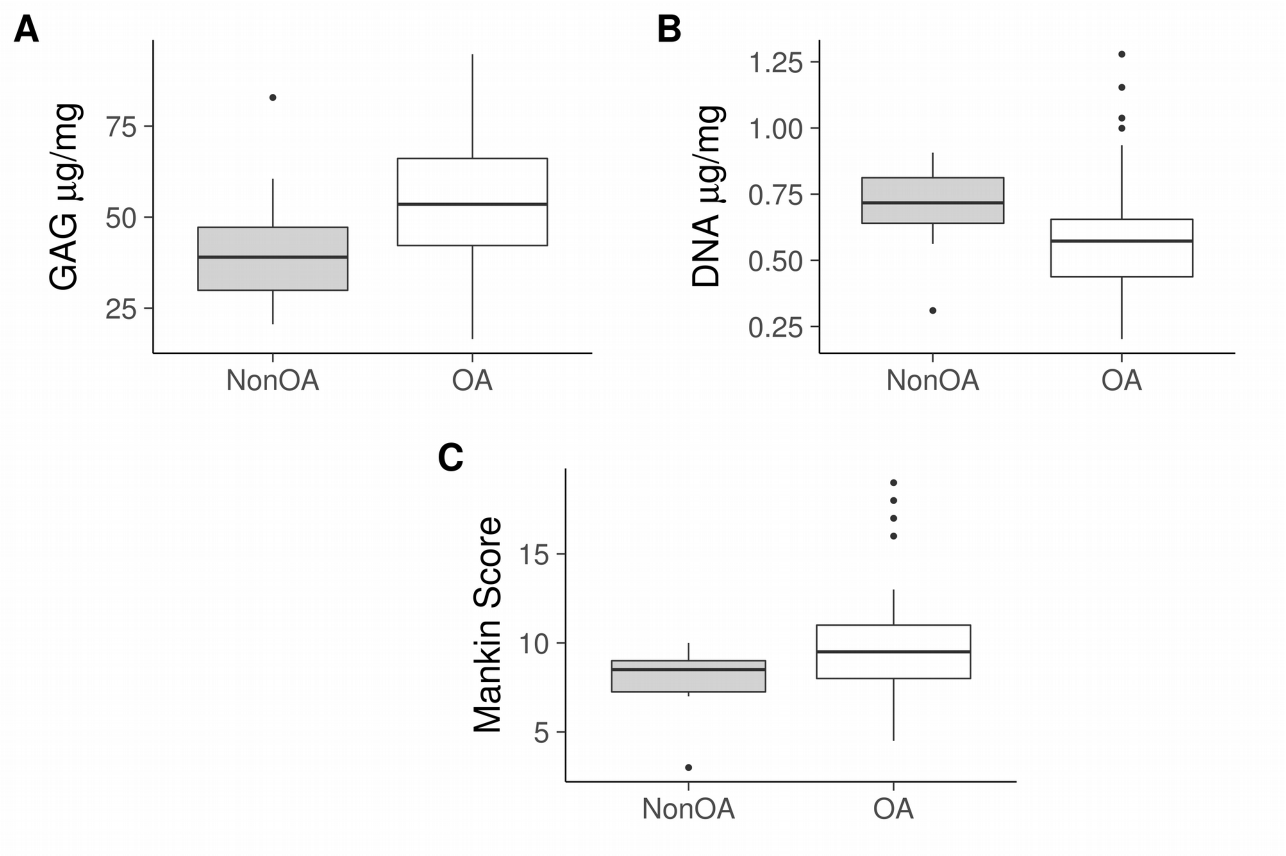

Supplementary Figure 2: OA Group patient statistics

The Age (**A**), Sex (**B**), BMI (**C**), Modified Mankin score of the articular cartilage (D), Pre-Operation (Pain) measured by VAS (**E**) and KOOS (Knee injury OA Outcome Score) (**F**) for OA Group A (n=24) and Group B (n=18).

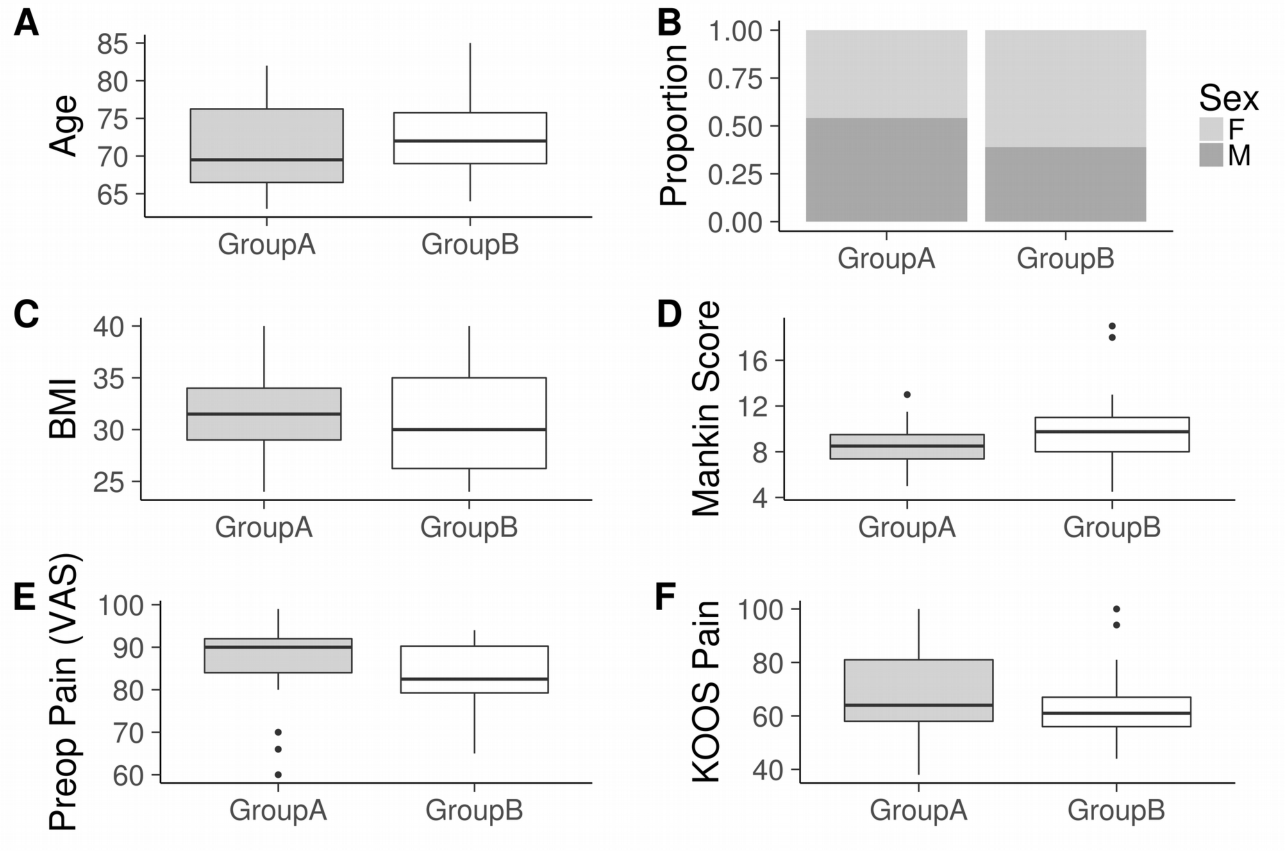

Supplementary Figure 3: Classifier gene panel

Boxplots showing the expression of the 10 gene classification panel in Group A and Group B in the discovery cohort for RNA-Seq (**A**) and RT-qPCR (**B**). **C** The RT-qPCR expression of the 10 gene panel in the validation cohort Group A (n=8) and Group B (n=8) as defined by the RNA-Seq classifier. Genes with (adjusted) p-value ≤0.05 are indicated with an asterisk.

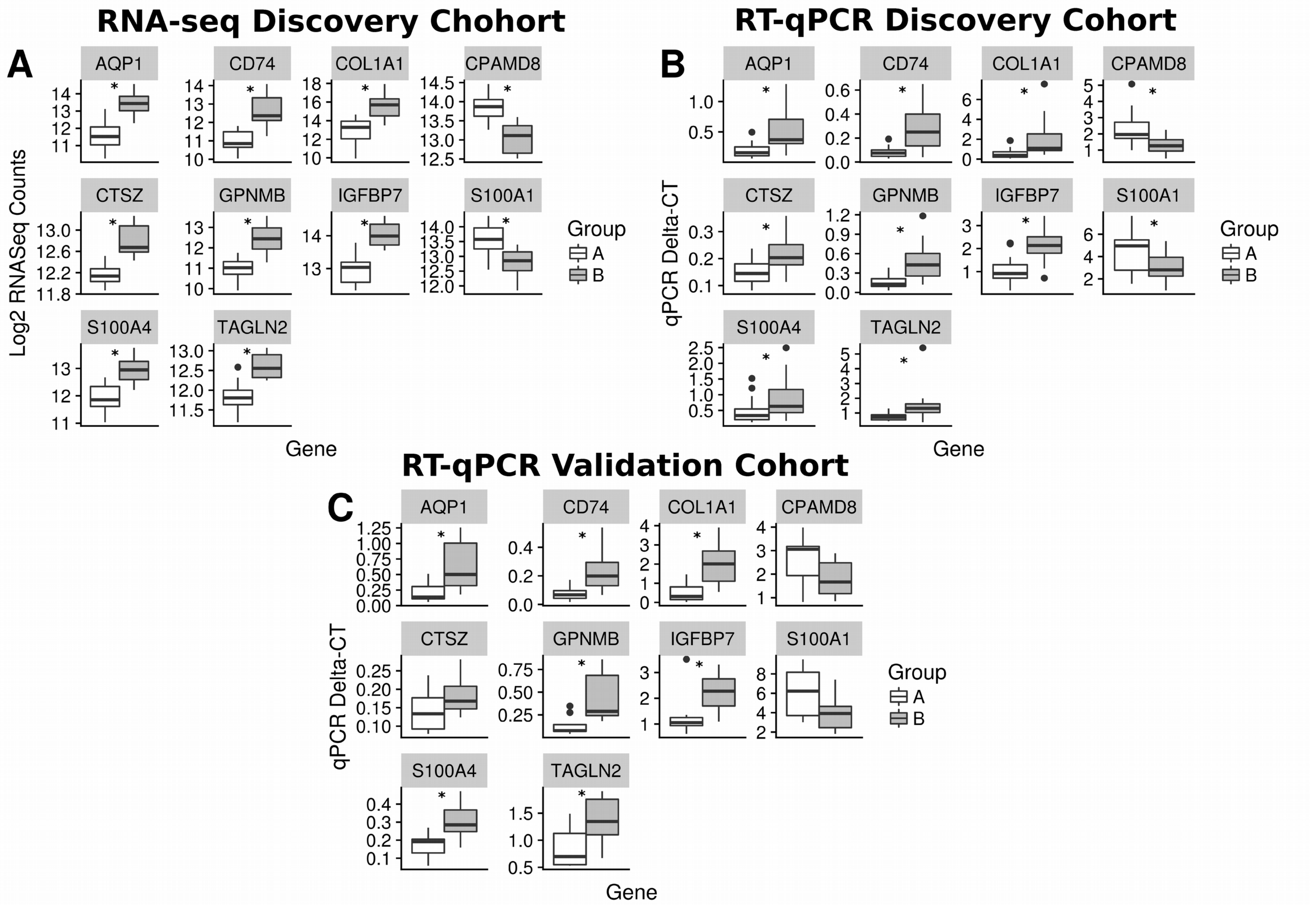

RNA-seq Discovery Chohort
RT-qPCR Discovery Cohort
RT-qPCR Validation Cohort
A
B
C
AQP1
CD74
COL1A1
CPAMD8
CTSZ
GPNMB
IGFBP7
S100A1
S100A4
TAGLN2
Log2 RNASeq Counts
qPCR Delta-CT
Gene
Group
A
B

Supplementary Figure 4: PhenomeExpress analysis of the OA subgroups

Differential gene expression data between OA Group A (n=32) and Group B (n=26) were analysed with the PhenomeExpress algorithm to identify dysregulated sub-networks using known disease gene associations. Nodes are coloured by fold change. Heatmaps show the gene expression between Group A, Group B and non-OA for the sub-networks.

Supplementary Figure 5: Expression in OA Group A and Group B of the gene panel proposed by Fernandez-Tajes et al 2014)

Expression of the set of 9 discriminative genes reported by Fernández-Tajes *et al.* in OA Group A (n=32) and Group B (n=26) was determined from the RNA-Seq results.

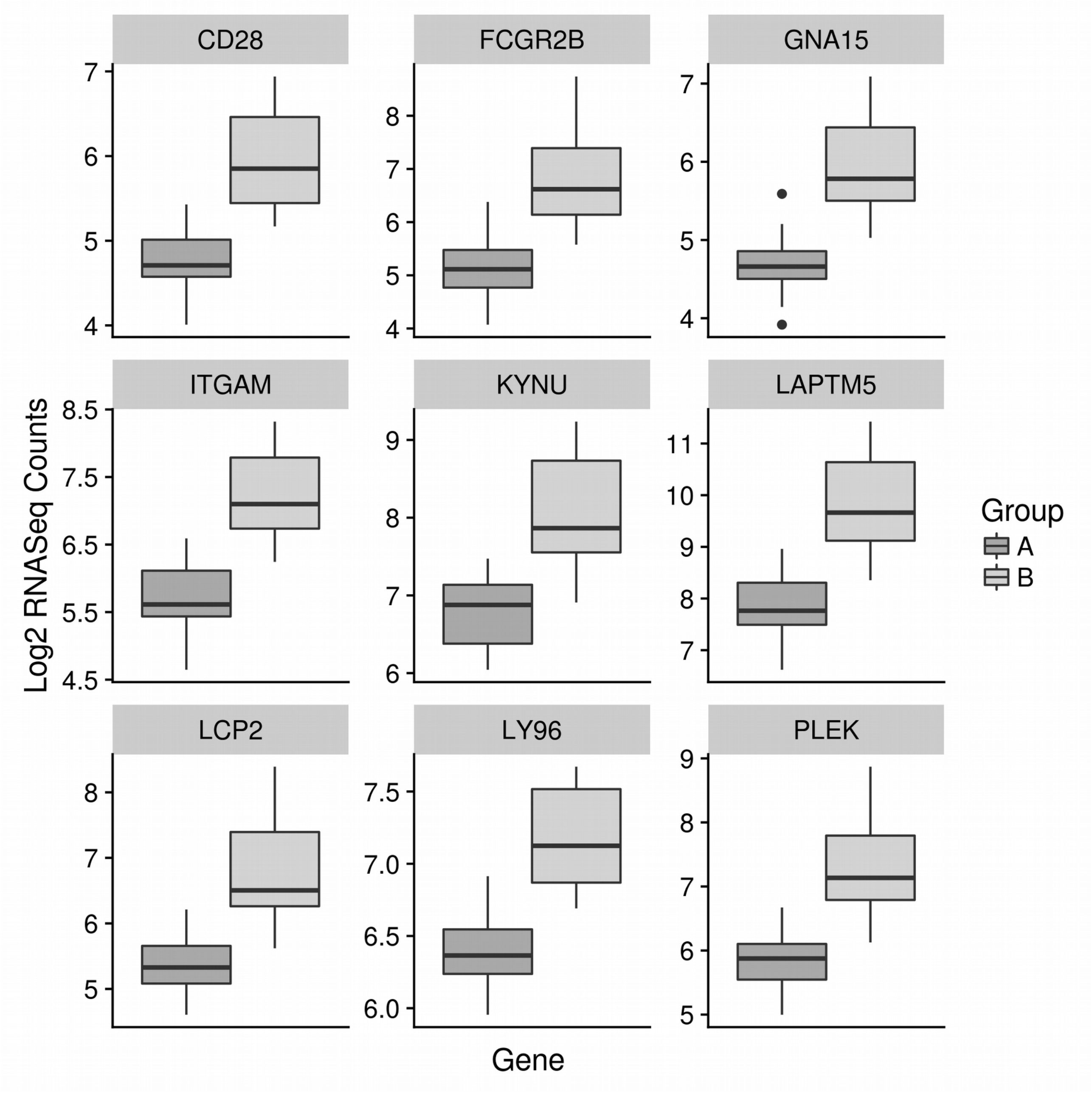

Supplementary Table 1: Differential expression between discovery cohort OA and non-OA patients

Differential gene expression between discovery cohort OA (n=42) and non-OA patients (n=10) was calculated using DESeq2. Differential expressed genes were defined as absolute fold-change ≥1.5 and ≤0.1 adjusted p-values.

Supplementary Table 2: Differential expression between all OA patients and non-OA patients

Differential gene expression between discovery cohort OA (n=58) and non-OA patients (n=10) was calculated using DESeq2. Differential expressed genes were defined as absolute fold-change ≥1.5 and ≤0.1 adjusted p-values.

Supplementary Table 3: Differential expression between OA Group A and Group B patients and non-OA patients

Differential gene expression between the combined discovery and validation cohort OA Group A(n=32), Group B (n=26) and non-OA patients (n=10) was calculated using DESeq2.

Supplementary Table 4: Reactome pathway enrichment between OA Group A and Group B and non-OA patients

Differentially regulated Reactome pathways between OA Group A, Group B and non-OA patients were identified with GOSeq. Signifiant pathways were defined by ≤0.05 adjusted p-values.

Supplementary Table 5: Matrix protein and associated genes differentially expressed between OA Group A and Group B

All genes listed are significantly changed between the two Groups with adjusted p-value ≤0.05 and for this comparison genes ≥ 1.3 fold change are included as many matrix genes have high expression and have highly significant, but low fold changes.

Supplementary Table 6: Summary of PhenomeExpress networks.

Differentially regulated sub-networks related to OA phenotypes. The size, empirical p-value and the function is indicated for each network.

Supplementary Table 7: Differential expression in Group A and Group B of genes identified by Fernandez-Tajes et al 2014

From the RNA-Seq results fold changes and p-values between OA Group A and Group B are shown for the set of 47 genes reported by Fernández-Tajes *et al.* 2014. These genes were reported to be significantly altered between their subgroups identified in independent DNA methylation and gene expression cohorts. Those genes differentially expressed in Group A and Group B were all regulated in the same direction as in the Fernandez-Tajes study.

Supplementary Table 8: Candidate biomarkers in synovial fluid to distinguish Group A and Group B patients

Proteins identified as differentially expressed (1.3 fold-change) between Group A (n=32) and Group B (n=26) which are secreted or released from chondrocytes and may be detected in synovial fluid.

Supplementary Table 9: RT-PCR primers

TaqMan primers used in RT-qPCR classifier gene panel.
