# Supplementary material for: Stratification of knee osteoarthritis: two major patient subgroups identified by genome-wide expression analysis of articular cartilage
Source: Ann Rheum Dis. 2017 Dec 22;77(3):423. doi: 10.1136/annrheumdis-2017-212603 (PMC5867416; doi:10.1136/annrheumdis-2017-212603)
Supplement: Supplementary file 6 [file annrheumdis-2017-212603supp006.docx]

| Gene | Protein | Group A | Group B |
| --- | --- | --- | --- |
| ACAN | Aggrecan | 1.56 |  |
| ADAMTSL2 | Matrix protein ADAMTS like2 | 1.56 |  |
| C4BPA | Complement 4 binding protein alpha | 1.73 |  |
| CHAD | Chondroadherin | 1.47 |  |
| CHADL | CHAD-like | 1.35 |  |
| CLEC3B | Tetranectin |  | 2.07 |
| CRTAC1 | Cartilage Acidic protein 1 |  | 1.55 |
| CYTL1 | Cytokine like 1, cartilage homeostasis | 1.36 |  |
| COL1A1 | Collagen1 alpha1 |  | 3.69 |
| COL1A2 | Collagen1 alpha2 |  | 1.92 |
| COL4A1 | Collagen4 alpha1 |  | 3.95 |
| COL4A2 | Collagen4 alpha2 |  | 3.20 |
| COL6A1 | Collagen6 alpha1 |  | 1.38 |
| COL6A2 | Collagen6 alpha2 |  | 1.42 |
| COL6A3 | Collagen6 alpha3 |  | 1.95 |
| COL9A1 | Collagen9 alpha2 | 1.39 |  |
| COL9A2 | Collagen9 alpha3 | 1.48 |  |
| COL14A1 | Collagen14 alpha1 |  | 1.89 |
| DKK3 | Dickkopf 3, Wnt inhibitor |  | 2.00 |
| ECM1 | ECM protein 1 |  | 1.77 |
| EMILIN1 | Elastin microfibril interfacer 1 |  | 1.87 |
| GPNMB | Osteoactivin |  | 2.73 |
| LAMA2 | Laminin A2 |  | 1.33 |
| LAMA4 | Laminin A4 |  | 1.66 |
| LAMB1 | Laminin B1 |  | 1.87 |
| LAMC1 | Laminin C1 |  | 1.32 |
| MGP | Matrix Gla protein | 1.48 |  |
| POSTN | Periostin |  | 2.80 |
| PRELP | Proline, arginine, leucine rich protein | 1.32 |  |
| SFRP1 | Secreted frizzled related protein 1 |  | 2.19 |
| SPARCL1 | Hevin, Ca++ binding |  | 3.11 |
| SPP1 | Osteopontin, bone sialoprotein |  | 1.66 |
| TGFBI | TGFbeta induced protein |  | 2.03 |
| THBS2 | Thrombospondin 2 |  | 1.57 |
| TIMP3 | Tissue inhibitor of metalloproteinase 3 |  | 1.67 |
| TNC | Tenascin C |  | 1.80 |
| TNFAIP6 | TSG6, TNF stimulated gene 6 |  | 1.99 |
| TNFSF11 | RANK ligand |  | 2.00 |
| VCAN | Versican |  | 2.40 |
| WIF1 | Wnt inhibitor factor 1 | 1.82 |  |
| WISP1 | Wnt inducible protein (CCN4) |  | 1.81 |
| WISP2 | Wnt inducible protein (CCN5) |  | 2.39 |
|  | TRANSCRIPTION FACTORS |  |  |
| RUNX2 | CBFA1 |  | 2.10 |
| SOX9 | SOX9 | 1.33 |  |
| SP7 | Osterix |  | 1.78 |

**Supplementary Table 5: Matrix protein and associated genes differentially expressed between OA Group A and Group B**

All genes listed are significantly changed between the two Groups with adjusted p-value ≤0.05 and for this comparison genes ≥ 1.3 fold change are included as many matrix genes have high expression and have highly significant, but low fold changes.
