# Supplementary material for: Stratification of knee osteoarthritis: two major patient subgroups identified by genome-wide expression analysis of articular cartilage
Source: Ann Rheum Dis. 2017 Dec 22;77(3):423. doi: 10.1136/annrheumdis-2017-212603 (PMC5867416; doi:10.1136/annrheumdis-2017-212603)
Supplement: Supplementary file 7 [file annrheumdis-2017-212603supp007.docx]

| Network | p-value | GO Term Function |
| --- | --- | --- |
| A | 0.004 | complement activation |
| B | 0.001 | Innate immune response |
| C | 0.001 | TLR4 signalling |
| D | 0.001 | Wnt signalling |
| E | 0.001 | Oxidative stress |
| F | 0.004 | T cell costimulation |
| G | 0.006 | Sensory perception of pain |
| H | 0.021 | Lipoxin metabolic process |
| I | 0.005 | MMP9 signaling |
| J | 0.001 | Negative regulation of GTPase activity |
| K | 0.001 | Apoptosis |
| L | 0.001 | Fc-epsilon receptor signaling pathway |
| M | 0.001 | Cell polarity |
| N | 0.011 | Negative regulation of calcium ion import |
| O | 0.009 | Receptor internalization |
| P | 0.001 | Complement activation |
| Q | 0.003 | Regulation of protein transport |
| R | 0.002 | IGF signaling |
| S | 0.015 | Cell adhesion |
| T | 0.001 | Heterotypic cell-cell adhesion |
| U | 0.003 | Chemokine-mediated signaling pathway |
| V | 0.001 | Positive regulation of cholesterol efflux |

**Supplementary Table 6: Summary of PhenomeExpress Networks (Fig 3, Suppl Fig4)**.

Differentially regulated sub-networks related to OA phenotypes. The size, empirical p-value and function is indicated for each network.
