# Supplementary material for: Stratification of knee osteoarthritis: two major patient subgroups identified by genome-wide expression analysis of articular cartilage
Source: Ann Rheum Dis. 2017 Dec 22;77(3):423. doi: 10.1136/annrheumdis-2017-212603 (PMC5867416; doi:10.1136/annrheumdis-2017-212603)
Supplement: Supplementary file 8 [file annrheumdis-2017-212603supp008.docx]

| gene_name | EnsemblID | log2FoldChange | padj |
| --- | --- | --- | --- |
| LAPTM5 | ENSG00000162511 | 2.073637969 | 5.26318E-12 |
| CD84 | ENSG00000066294 | 2.407976007 | 1.30609E-11 |
| MSR1 | ENSG00000038945 | 1.933188383 | 1.99897E-10 |
| LILRB5 | ENSG00000105609 | 1.976145862 | 1.24166E-09 |
| CD86 | ENSG00000114013 | 1.990582397 | 2.20651E-09 |
| PECAM1 | ENSG00000261371 | 2.201179554 | 2.27476E-09 |
| NCKAP1L | ENSG00000123338 | 1.520472607 | 2.30079E-09 |
| IGSF6 | ENSG00000140749 | 1.990380444 | 3.49896E-09 |
| CYTIP | ENSG00000115165 | 1.898813144 | 1.25251E-08 |
| LCP1 | ENSG00000136167 | 1.947348621 | 1.3626E-08 |
| NCF4 | ENSG00000100365 | 2.238032499 | 1.61823E-08 |
| NCF2 | ENSG00000116701 | 1.804475116 | 2.69097E-08 |
| SELPLG | ENSG00000110876 | 1.945186093 | 4.10476E-08 |
| KYNU | ENSG00000115919 | 1.561079213 | 4.25857E-08 |
| PLEK | ENSG00000115956 | 1.688460386 | 5.60932E-08 |
| FERMT3 | ENSG00000149781 | 1.425222865 | 6.33927E-08 |
| CTSZ | ENSG00000101160 | 0.646008855 | 1.19496E-07 |
| CD28 | ENSG00000178562 | 1.864592688 | 1.38237E-07 |
| GNA15 | ENSG00000060558 | 1.90652634 | 1.38237E-07 |
| MARCO | ENSG00000019169 | 1.895416353 | 3.04662E-07 |
| FCGR2B | ENSG00000072694 | 1.954638798 | 3.13195E-07 |
| LCP2 | ENSG00000043462 | 1.847800477 | 3.13195E-07 |
| FCER1A | ENSG00000179639 | 2.205858093 | 7.73987E-07 |
| ITGAM | ENSG00000169896 | 1.542562807 | 8.94063E-07 |
| CD33 | ENSG00000105383 | 1.703271093 | 5.40963E-06 |
| LST1 | ENSG00000231048 | 1.552093562 | 2.37948E-05 |
| MX2 | ENSG00000183486 | 0.90130432 | 5.25215E-05 |
| AMT | ENSG00000145020 | -0.548652439 | 7.10055E-05 |
| KCNMB1 | ENSG00000145936 | 0.878806959 | 0.000204842 |
| SIGLEC10 | ENSG00000142512 | 0.946996778 | 0.000639764 |
| ITGAX | ENSG00000140678 | 1.308786695 | 0.001056232 |
| TREM1 | ENSG00000124731 | 1.13845988 | 0.002063058 |
| CRIM1 | ENSG00000150938 | -0.34983232 | 0.014921967 |
| DDAH1 | ENSG00000153904 | -0.290901734 | 0.015647872 |
| IL16 | ENSG00000172349 | -0.310974151 | 0.017834141 |
| GPR75 | ENSG00000119737 | -0.347317419 | 0.028316677 |
| SLC44A2 | ENSG00000129353 | -0.352796609 | 0.031711601 |
| DCC | ENSG00000187323 | -0.5708632 | 0.032405328 |
| DDR2 | ENSG00000162733 | -0.251150987 | 0.033724416 |
| ABCA5 | ENSG00000154265 | -0.348145315 | 0.034168473 |
| CCL28 | ENSG00000151882 | -0.21643592 | 0.049111515 |
| RHOJ | ENSG00000126785 | -0.244029773 | 0.069209134 |
| CYGB | ENSG00000161544 | -0.209110218 | 0.168242458 |
| PLSCR4 | ENSG00000114698 | -0.092410245 | 0.320851689 |
| HLA-DPB1 | ENSG00000223865 | 0.506117473 | 0.392166132 |
| ZNF134 | ENSG00000213762 | 0.055721109 | 0.631198842 |
| PLA2R1 | ENSG00000153246 | -0.051379288 | 0.776367384 |

Supplementary Table 7: Differential expression in Group A and B of genes identified by Fernandez-Tajes et al 2014

From the RNA-Seq results fold changes and p-values between OA Group A and Group B are shown for the set of 47 genes reported by Fernández-Tajes *et al.* 2014. These genes were reported to be significantly altered between their subgroups identified in independent DNA methylation and gene expression cohorts. Those genes differentially expressed in Group A and Group B were all regulated in the same direction as in the Fernandez-Tajes study.
